# Galectin Targeted Therapy in Oncology: Current Knowledge and Perspectives

**DOI:** 10.3390/ijms19010210

**Published:** 2018-01-10

**Authors:** Kamil Wdowiak, Tomasz Francuz, Enrique Gallego-Colon, Natalia Ruiz-Agamez, Marcin Kubeczko, Iga Grochoła, Jerzy Wojnar

**Affiliations:** 1 Department of Internal Medicine and Oncology, Silesian Medical University, Katowice 40-027, Poland; wdowiak.kamil@op.pl (K.W.); marcin.kubeczko@gmail.com (M.K.); groc.iga@gmail.com (I.G.); jwojnar@sum.edu.pl (J.W.); 2 Department of Biochemistry, Silesian Medical University, Katowice 40-752, Poland; enrique.gce@gmail.com (E.G.-C.); natalia.ruiz.agamez@gmail.com (N.R.-A.); 3 Clinical and Experimental Oncology Department, Maria Skłodowska-Curie Memorial Cancer Center and Institute of Oncology, Gliwice Branch, Gliwice 44-101, Poland

**Keywords:** galectins, cancer, diagnosis, galectins in therapy

## Abstract

The incidence and mortality of cancer have increased over the past decades. Significant progress has been made in understanding the underpinnings of this disease and developing therapies. Despite this, cancer still remains a major therapeutic challenge. Current therapeutic research has targeted several aspects of the disease such as cancer development, growth, angiogenesis and metastases. Many molecular and cellular mechanisms remain unknown and current therapies have so far failed to meet their intended potential. Recent studies show that glycans, especially oligosaccharide chains, may play a role in carcinogenesis as recognition patterns for galectins. Galectins are members of the lectin family, which show high affinity for β-galactosides. The galectin–glycan conjugate plays a fundamental role in metastasis, angiogenesis, tumor immunity, proliferation and apoptosis. Galectins’ action is mediated by a structure containing at least one carbohydrate recognition domain (CRD). The potential prognostic value of galectins has been described in several neoplasms and helps clinicians predict disease outcome and determine therapeutic interventions. Currently, new therapeutic strategies involve the use of inhibitors such as competitive carbohydrates, small non-carbohydrate binding molecules and antibodies. This review outlines our current knowledge regarding the mechanism of action and potential therapy implications of galectins in cancer.

## 1. Introduction

The carbohydrate alphabet acts as second genetic code containing necessary information to carry out many of cellular processes. The “sugar code”, in the case of glycans has become immensely complex and creates a vast number of “word” combinations, which translate into bioactive information that triggers specific effects. This “sugar code” is translated inside the cell by sugar-binding proteins called lectins. Galectins are a subfamily of lectin proteins with high affinity for β-galactosides. In normal tissue and blood, galectins are expressed at low levels, but they are increased in serum, plasma and urine in neoplastic diseases [1]. Interestingly, galectins also play an important role in other chronic diseases such as cardiac insufficiency, diabetes, rheumatoid arthritis, asthma and liver cirrhosis [2]. The basic domain of galectins contains a carbohydrate recognition domain (CRD) through which they can bind with numerous carbohydrate ligands. To date, up to 16 members of the galectin family have been discovered in mammals, 12 of which have been identified in humans.

Depending on their structure, galectins may be divided into three groups: prototype, tandem repeats and chimeric galectins (Table 1) [3].

Galectins are present in numerous locations within the cell, such as nucleus, cytoplasm and plasma membrane, but also extracellularly. The distinct glycosylation of glycoproteins allows binding of galectins to β-galactosides in different areas of the protein, leading to prolonged receptor activation at the plasma membrane. Given that glycosylation is the most frequent post-translational modification, galectin–proteoglycan interactions might be an important phenomenon. In cancer, continuous stimulation of VEGFR2 promotes the formation of new blood vessels and thus facilitating cancer progression and metastasis [4]. Current therapies involve the use of vascular endothelial growth factor (VEGF) inhibitors such as Bevacizumab. Patients treated with VEGF-targeted therapies showed varying efficacies and tumor regrowth. Croci and collaborators found that Gal-1 can recognize *N*-glycans on VEGFR2 and trigger a VEGF-like signaling response thereby promoting vascular regrowth in absence of VEGF [4]. Additionally, galectins are involved in cancer-promoting processes such as proliferation, apoptosis and immune modulation. Galectins produced by tumor cells bind to T-cell glycoprotein receptors like CD45 and CD71 [5]. In particular, extracellular Gal-1 and extracellular Galectin-3 (Gal-3) have been implicated in promoting T-cell suppression and apoptosis, while intracellular Gal-3 promotes activation of anti-apoptotic pathways in T-lymphocytes [4,5].

The design of selective inhibitors for galectins is challenging because of the shared homology of the CRDs among lectins, which can range from 20% to 50% [6]. Additionally, tumor cells can generate multiple isoforms via alternative splicing and this can result in inhibitor-resistant galectins [1]. Furthermore, even if selective inhibition is achieved, other galectins can compensate for the inhibited type. This effect was observed for Gal-1 and Gal-3 in pancreatic cancer cells. The compensatory mechanism involves p16^INK4a^, a tumor suppressor that inhibits cyclin-dependent kinases; p16^INK4a^ modulates and affects the reactivity and expression of lectins by downregulating Gal-3 levels. In this case, compensatory increased Gal-1 extracellular levels were observed. Intracellular Gal-3 downregulation caused reduction of the anti-apoptotic effect [7]. Potential adverse effects of galectin inhibition were also observed in human breast carcinoma. In breast cancer cells, Gal-1 and Gal-3 compete for cell surface receptors while generating opposite functions. Gal-3 binds with K-Ras and activates the MEK-ERK signaling pathway, while Gal-1 binds with H-Ras and activates PI3K/AKT cascade hence modulating rather distinct cellular functions [8].

## 2. Galectin-1

In healthy tissues, Gal-1 is located inside the cell, the cytoplasm, or nucleus [9] and remains there until cell activation [10]. Gal-1 secretion into the extracellular matrix (ECM) also occurs to a lesser extent. Increased expression of Gal-1 is observed in numerous neoplasms, including colorectal [11], lung [12], breast [13], pancreas [14,15], liver [16], thyroid [17] and hematological malignancies [18,19]. Additionally, an increase in Gal-1 blood concentration was observed in lung cancer [12], thyroid cancer [20], T cell lymphoma [21] and glioma [22].

Gal-1 can act at intracellular level as an effector of pre-mRNA splicing, or extracellularly as a binding protein to numerous glycoproteins, glycolipids and elements of the extracellular matrix (ECM) [23]. Consequently, Gal-1 has the potential to affect adhesion and aggregation of cells, especially in neoplastic cells where it can influence metastatic processes [24]. Gal-1 binding proteins have been identified including integrins, laminins, fibronectin, thrombospondin, vitronectin, osteopontin, neuropilin-1 (NRP-1), CD44, CD146 and CD326 [24]. Paz and colleagues suggested additional roles for Gal-1. Intracellular Gal-1 reacts with the active form of oncogenic H-Ras (H-Ras-GTP), thereby increasing its membrane anchorage, a crucial step in malignant transformation of certain cancers [25]. Gal-1 has also been associated with immunosuppression, stimulating apoptosis in activated T CD4+ and CD8+ lymphocytes [26]. Indeed, Gal-1 targeted therapy may contribute to reduce the dissemination of tumor cells and inhibit angiogenesis and tumor growth.

Currently, anti-angiogenic treatments have therapeutic limitations such as varying degrees of response and resistance. This phenomenon is thought to occur due to VEGF-independent mechanisms. In hypoxic areas, tumor cells survive oxygen-depleted environment by up-regulating the expression of hypoxia-inducible factor-1 (HIF-1α) [27]. Studies show that colorectal cancer cell lines cultured in hypoxic environment produce larger amounts of Gal-1, which correlated with increased hypoxic factors such as hypoxia induced factor α (HIF-1α) as well as carbonic anhydrase IX (CAIX) [28,29]. The studies described in this section suggest that inactivation of Gal-1 in tumor cells may result in an increased sensitivity to chemotherapeutic agents.

Several Gal-1 inhibitors have been designed with potential clinical application in cancer therapy.

### 2.1. Thiodigalactoside

Thiodigalactoside or TDG is a synthetic disaccharide with affinity for Gal-1. TDG non-selectively blocks Gal-1 action during angiogenesis and immune response and protects against oxidative stress (Table 2). Intra-tumoral treatment with TDG suppresses growth of breast cancer and melanoma in preclinical models [30]. Interestingly, the influence of TDG in blocking tumor progression was not observed in Gal-1 knock-out mice, indicating that Gal-1 is a TDG target. In a preclinical study, Gal-1 knock-out mice showed an increase in T CD4+ and CD8+ lymphocytes in the tumor milieu, in blood and in immunocompetent organs. An effect of TDG on angiogenesis was evidenced by the reduction in number of endothelial cells (CD31+) and in new vessel formation [30]. In subsequent studies, Ito and colleagues observed that after TDG administration, the number and size of lung metastases of mice carrying breast or colon tumors was decreased. The mechanism of action of TDG is by preventing binding of Gal-1 to CD44 and CD326 receptors on the surface of cancer stem cells (CSC) [30,31,32].

### 2.2. Anginex (β Pep-25)

Anginex is an antiangiogenic peptide involved in tumor growth (Table 2) [33,47]. The drug contains short sequences of known antiangiogenic factors such as platelet factor-4 (PF4), interleukin 8 (IL-8) and bactericidal-permeability increasing protein-1 (BPI-1) [48]. Anginex, specifically binds to the β-sheet motif of Gal-1, inhibiting neoplastic proliferation, migration and inducing apoptosis, thereby inhibiting tumor growth [49]. This drug also blocks Gal-1 uptake by endothelial cells, thereby preventing the translocation of H-Ras-GTP and phosphorylation of the Raf/MEK/ERK kinase cascade [50].

Numerous clinical studies evaluated the effect of Anginex in combination with radiotherapy and/or chemotherapy. The results showed that Anginex sensitizes tumor-associated endothelial cells to radiotherapy, thus strengthening the therapeutic effect [34,49,50,51,52,53]. In a human ovarian carcinoma mouse model, Anginex showed synergistic effect with a suboptimal dose of Carboplatin and boosted tumor regression [54]. Amano and colleagues showed that Anginex is able to prolong radiation-induced tumor regression in a squamous cell xenograft model [51]. Furthermore, several studies have focused on increasing Anginex bioavailability by modifying the structure and/or by conjugation with carrier proteins to increase treatment efficacy [36,55,56,57]. A very innovative and interesting study by Upreti and colleagues showed that Gal-1 is overexpressed in triple negative breast cancer (TNBC) relative to patients with normal tissue or benign breast lesions. They developed a murine model of TNBC, with radiation-induced Gal-1 expression in stromal tissue. Complexes of Anginex and arsenic trioxide, as well as Cisplatin-loaded liposomes were tested this model, leading to decreased tumor growth by ~80% (vs. 20% in non-irradiated mice treated with non-conjugated liposomes) [58]. Indeed, Anginex nanotherapy is a well-tolerated, very effective therapy with potential application as Gal-1 overexpression occurs in approximately eight to ten samples of ductal breast carcinoma human tissue. Interestingly, new Anginex analogues such as Dibenzofuran (6DBF7), DB16 and DB21 also showed similar results with an 80% reduction in tumor growth following administration in mice [37]. In particular, DB21 appears to inhibit angiogenesis and tumor growth very effectively [49,59]. Anginex’s beneficial effects on tumor suppression are antagonized by its reduced stability and half-life, difficulty in manufacture and that it only sensitizes endothelial cells to radiotherapy in newly forming tumor vessels, not in tumor cells. Anginex interacts with other galectins, such as Gal-2, -7, -8N and 9N, but with lower affinity [38]. To conclude, Anginex therapy shows minimal side effects in Anginex-treated animal models, either alone or in combination with chemotherapy or radiotherapy [39,51,58].

### 2.3. OTX008 (0018)

OTX008 is chemically more stable and resistant to hydrolysis when compared to other Gal-1 inhibitors (Table 2). The low molecular weight (937 Da) and the fact that it is neither a protein nor saccharide, but a phenyl-based molecule greatly increases its bioavailability [39]. From a mechanistic point of view, OTX008 binds Gal-1 at a more distant location within the CRD as compared to Anginex. Additionally, OTX008 has both direct and indirect effects on cell survival, cell cycle and angiogenesis. Research has shown that administration of OTX008 in vivo and in vitro is effective, both in single and combination therapies [40,41,42]. Astorgues-Xerri et al. evaluated the efficiency of OTX008 in several cancer cell lines and in a murine ovarian carcinoma model [60]. Ovarian cancer cells of epithelial origin were shown to be more sensitive to OTX008 than cells of mesenchymal origin. Moreover, OTX008 exposure inhibited p-ERK 1/2 and p-AKT survival signaling pathways. OTX008 also caused G_2_/M cell cycle arrest by modulating the activity of CDK1 via G_2_/M checkpoint-regulators CDC25 and WEE1. In vivo experiments showed OTX008 inhibits tumor growth, accompanied by a decreased in Gal-1, Ki67 and VEGFR2 expression. Synergistic activity with other chemo- or immunotherapies was also achieved in in vitro therapies using drugs such as Cisplatin, Oxaliplatin, Docetaxel, 5-fluorouracil, Regorafenib, Sunitinib and Everolimus [55]. Combination treatment of mTOR inhibitor Rapamycin and OTX008 was more effective than Rapamycin alone in limiting tumor volume and reducing the number of cells with HRAS mutation [61]. In 2012, a phase I clinical trial aimed at evaluating the effects of subcutaneous administration of OTX008 for the treatment of advanced solid tumors (ClinicalTrials.gov: NCT01724320) [24]. However, so far, no results regarding outcome of treatment have been released. Recently, a Calixarene-based topomimetic of OTX008, PTX013, showed improved efficiency and greater potency than OTX008. Preliminary data indicates that PTX013 actions are not directed at Gal-1 and the molecular target is yet to be found [62]. Additionally, the possibility of combinatorial treatments with chemotherapeutics is an intriguing option that is being explored [43].

### 2.4. F8.G7

Interaction of Gal-1 and VEGFR2 leads to prolonged presence of the receptor in the cell membrane of endothelial cells thereby promoting tumor regrowth, which may limit the efficacy of anti-VEGF treatment [4]. Croci Do et al. showed that monoclonal anti-Gal-1 (F8.G7) (Table 2) based therapy inhibited tumor growth and angiogenesis, including pathways associated with VEGFR2/Gal-1 in mice with Kaposi’s sarcoma [44]. Vessels of treated tumors decreased in size and number, were less dispersed and covered with mature pericytes. Additionally, increased T lymphocyte infiltration and production of IFN-γ and IL-17 was observed. Importantly, F8.G7 therapy only targets the non-canonical VEGF pathway and the canonical pathway can still contribute to tumor angiogenesis. As concluded by these researchers, further research is required and personalized therapy should be the aim of treatment.

### 2.5. GM-CT-01 (DAVANAT^®^) oraz GR-MD-02

GM-CT-01 (DAVANAT^®^) is a modified vegetal galactomannan oligomer extracted from Guar seeds (*Cyamopsis tetranoglonoloba*) (Table 2)*.* Davanat shows affinity to the dimer interface rather than the CRDs in Gal-1 and Gal-3 [45]. Demotte et al. reported improved tumor infiltrating lymphocyte (TIL) function induced by GM-CT-01 [63]. Extracellular Gal-1 and Gal-3 are responsible for blockade of glycosylated receptors on the surface of TILs leading to reduced T-cell motility and overall function. Galactomannan treatment promotes IFN-γ secretion by T-cells, which promotes an antitumor response. GM-CT-01 therapy progressed into phase I and II clinical trials for the treatment of solid tumors. Unfortunately, the trials were prematurely terminated due to financial reasons, nevertheless a certain degree of therapeutic effect was observed in patients suffering from metastatic colorectal cancer (mCRC). In the DAVANAT^®^ trial (NCT: NCT00054977), out of 20 subjects enrolled, one had a partial response to the drug while six other patients had stable disease. Moreover, lower frequency of 5-Fluorouracil (5-FU) side effects for grades 3–4 (G3–G4) was seen in combined treatment with GM-CT-01 [46]. At present, an ongoing phase II clinical trial is being conducted using a GM-CT-01 vaccine in patients suffering from diffuse melanoma (NCT: NCT01723813). In preclinical models, Gal-1 facilitates the escape of melanoma cells from immune surveillance by reducing the number of helper T-cells and cytolytic T-cells [64]. Downregulation of Gal-1 by siRNA knockdown in B16F10 cell lines resulted in an increase in response rates to Temozolamide and increased survival time of B16F10 melanoma-bearing mice [65]. In a recent study, Wu and colleagues observed that patients treated with Bevacizumab (anti-VEGF antibody) and Ipilimumab (anti-CTLA-4 antibody) that also received anti-Gal-1 antibody had a longer overall survival (OS). In contrast patients with higher Gal-1 levels had shorter OS [66]. Inhibition of Gal-1 functions may enhance the activity of checkpoint inhibitors and restore T-cell activity.

Additionally, a modified version of the DAVANAT^®^ drug, GR-MD-02, proved to be effective in the treatment of non-alcoholic steatohepatitis (NASH) in mice [67]. Reduction of inflammation, fat accumulation, fibrosis and hepatocellular damage were observed. In the randomized phase I study, no serious adverse events were observed with GR-MD-02 at doses of 2, 4 and 8 mg/kg [68]. In advanced stages of melanoma Gal-3 is overexpressed and its serum concentration increases [69,70,71]. Currently, two more clinical trials are being conducted using GR-MD-02 in combination with Ipilimumab or Pembrolizumab in patients suffering from melanoma (NCT: NCT02117362 and NCT02575404).

## 3. Galectin-3

Galectin-3 (Gal-3) is the only representative of the chimeric galectin group. Gal-3 is composed of a collagen-like sequence, a C-terminal domain (CTD) with a CRD, an N-terminal domain (NTD) with a serine phosphorylation site. The CRD of Gal-3 contains 110–130 amino acids with NWGR motifs which are important for interaction with anti-apoptotic proteins of the Bcl-2 family [72]. The C-terminus is responsible, among other functions, for binding saccharides such as *N*-acetyllactosamine (LacNAc) and lactose. Furthermore, Gal-3 has a higher affinity to polysaccharides terminating in galactose than to monosaccharides. The CRD contains five subunits (A–E) among which the C subunit is responsible for recognizing glycans containing β-galactosides [73]. The NTD facilitates multimerization and pentamer formation of galectin-3, which is necessary for extracellular secretion and nuclear translocation [74]. Based on the Gal-3 crystallographic structure, a number of low molecular weight and high affinity inhibitors have been developed [75]. A fraction of these compounds such as TDG and derivatives are currently being tested.

Gal-3 protein, present in both healthy tissues and neoplastic tissues, is involved in processes such as inflammation, neoplasia, cancer cell adhesion, angiogenesis, cell growth, proliferation and apoptosis [76]. A correlation between Gal-3 and such processes was shown for thyroid, stomach, large intestine, kidney, lung, prostate, breast and pancreatic cancers [1]. Importantly, immunohistochemical (IHC) staining of Gal-3 protein can provide a useful diagnostic tool for the differentiation of benign and malignant thyroid nodules, as demonstrated in several studies [77,78,79]. Based on a meta-analysis of 52 studies, the sensitivity and specificity of Gal-3 IHC expression was 87% and 87%, respectively [80]. Depending on its intracellular location, Gal-3 may have pro- or anti-apoptotic effects. In the nucleus, Gal-3 is responsible for gene expression regulation through transcription factors such as SP1 and β-catenin. It also plays a role in micro-RNA expression and splicing, as well as in transport of nuclear proteins [81,82,83]. In the cytoplasm, Gal-3 modulates numerous signaling pathways involved in cancer such as RAS, BCL-2 and MYC [84,85,86]. Moreover, this lectin is responsible for dampening the immune response through suppression of T-cells and natural killer (NK) cells and to induce apoptosis of T-cells by binding CD45 [87,88]. Additionally, endogenous Gal-3 may inhibit Cisplatin- or Etoposide-induced mitochondrial apoptosis pathway in prostate and breast cancer cells [84,89]. The role of Gal-3 in antineoplastic-resistant treatment is noteworthy and therefore its inhibition may be key in overcoming resistance and increase susceptibility of neoplastic cells to drugs [90]. Recently, Harazono et al. showed that extracellular Gal-3 takes part in a previously unknown chemoresistance mechanism [91] by which Gal-3 increases activity of Na/K ATPase. Following an application of Gal-3 inhibitor GCS-100, an increase in sensitivity to Doxorubicin was observed in tumor cells [92].

### 3.1. G3–C12

G3–C12 is an oligopeptide that binds Gal-3 at the CRD region. In a mouse model of breast cancer, mice subjected to G3–C12, had decreased metastasis formation (Table 3) [93]. Yang et al. were the first to use a conjugate therapy composed of a G3–C12 and 5-Fluorouracil, P-(G3–C12)-FU, on a mouse model of prostate cancer [94]. In this research, G3–C12 was also complexed with *N*-(2-hydroxypropyl) methacrylamide (HPMA) as a carrier molecule. Enhanced drug delivery was observed due to the low molecular weight of the HMPA compound, which facilitates delivery inside the cell. This complex yielded far better therapeutic results than 5-Fluorouracil (5-FU) therapy alone. Furthermore, G3–C12–HPMA conjugate shows better pharmacokinetics and bioavailability than with the chemotherapeutic agent [95]. Subsequently, Doxorubicin (DOX) or 5-FU was added to the HPMA/G3–C12 complex [96]. In vivo P-(G3–C12)-DOX-FU showed the strongest effect among other combinations and inhibited tumor growth in mice by 81.6%, whereas the other agents were less effective (P-DOX-FU—71.2%, P-DOX—63%, DOX-HCl—40.5%, P-FU—32%, 5-FU—14.6%). Current studies aim to identify how the copolymer binds the cell [97]. Researchers hypothesized that facilitated by G3–C12, the drug conjugate can bind Gal-3 and become internalized. Besides, the presence of DOX also leads to translocation of Gal-3 into mitochondria triggering antiapoptotic effects. However, progressive inflow of Gal-3 promotes drug accumulation in the mitochondria. With time, Gal-3 function is inhibited, while mitochondria dysfunction is exacerbated by the activity of DOX. Consequently, G3–C12 holds enormous potential, however, the mechanism of action is unknown.

### 3.2. Modified Citrus Pectins (MCP)

Modified citrus pectins (MCP) are a group of polysaccharides derived from citrus fruits, which have been subject to chemical or thermal modification. MCPs such as Pecta-Sol and GCS-100, inhibit Gal-3 function and ligands such as cytokines or type C lectins [110,111]. MCP antineoplastic actions include tumor growth suppression by halting cell cycle, apoptosis activation, sensitization of tumor cells to chemotherapy, reduction of metastatic and angiogenesis potential and restoration of immune function (Table 3).

In a study that included 26 patients with various solid tumors, a hydrolyzed form of MCPs was orally administered at a dosage of 5 grams three times a day. After two cycles (eight weeks) of treatment, 11 patients (42.3%) had achieved stable disease and six patients (23.0%) maintained stable disease status for at least 24 months [101]. These results should be weighted considering that the subjects had advanced and aggressive tumors. Additionally, the purpose of the study was to assess tolerability, quality of life and clinical benefit response. Other citrus pectins with higher affinity for Gal-3 are being investigated and already existing pectins are being modified with the aim of increasing their antineoplastic potential.

#### 3.2.1. PectaSol-C

Yan and collaborators observed that the MCP family member PectaSol-C, inhibited tumor growth by blocking the MAPK cascade in prostate cancer cell lines. Effectiveness was assessed in prostate cancer cell lines, which showed a halt in proliferation and induction of apoptosis [98]. Later studies showed synergism of PectaSol-C with Doxorubicin in prostate cancer and with Paclitaxel in ovarian cancer cell lines [103,104]. In both cases tumor size was decreased (Table 3). Currently, a Phase III (NCT: NCT01681823) clinical trial is being carried out to test whether oral administration of PectaSol-C can improve prostate-specific antigen (PSA) kinetics in men with relapsed prostate cancer. In another study, MCP-treated HUVEC cells lost motility and cellular organization [99], tumors decreased in size and angiogenesis and growth of metastases were reduced [100].

#### 3.2.2. GCS-100

GCS-100 is a branched polysaccharide, synthesized from modified MCPs. This Gal-3 inhibitor induces apoptosis in multiple myeloma cells including resistant myeloma cells to Doxorubicin, Melfalan, Dexamethason [92] and Bortezomib [105] (Table 3). A similar effect was observed in prostate cancer cells, where Gal-3 inhibition by siRNA or administration of GCS-100 increased Cisplatin-induced apoptosis [106]. Downregulation of Gal-3 expression on the surface of diffuse large B-Cell lymphoma (DLBCL) cells sensitized them to immunochemotherapy [107].

On the basis of the latest reports, when combined with a BH-mimetic, GCS-100 induces apoptosis of acute myeloid leukemia (AML) cells, especially in cases with predominant negative prognostic factors, such as FLT3 ITD mutations. The effect of GCS-100 appears to be related to induction of p53, because cases where its expression was not induced or p53 was otherwise inactive, resulted in no response to treatment [108]. Based on these studies, it can be concluded that GCS-100 is a good candidate, however, more research and larger studies are required to determine its efficacy. In a phase II clinical trial, 24 patients with recurrent chronic lymphocytic leukemia, were treated with GCS-100 intravenously at a dose of 160 mg/m^2^ in a 5-day regiment, every 21 days. In 6 patients (25%) partial response was observed and the disease was stable in 12 patients (50%) [109]. Good overall tolerance was observed, with only minor complications such as nausea, skin rash and low to moderate fatigue. In addition to the antineoplastic roles of GCS-100, Gal-3 inhibition modulates immune system response. Demotte et al. explored the role of Gal-3 in immune system function. Inhibition of Gal-3 with GCS-100 in mice resulted in restoration of CD8+ and CD4+ T cell function. An increase in IFN-γ secretion by TILs was also observed along with tumor regression [102]. In a review by Zhang et al., the mechanism of action and anti-cancer properties of MCPs are discussed [112]. Unfortunately, limited reports on the application of MCPs in patients suffering from neoplasms are available.

## 4. Galectin-4

Galectin-4 (Gal-4) contains C- and N-terminal CRDs that share 38% amino acid sequence similarity. Two Gal-4 CRDs with different binding partners are connected by a linker region. Gal-4 is a tandem-repeat galectin expressed in epithelial cells of gastrointestinal tract [113]. To date, our knowledge on Gal-4 is restricted to the differences in expression observed in healthy versus cancerous tissues. Gal-4 is considered a risk factor for lymph node involvement in lung cancer [114]. Moreover, high levels of Gal-4 are seen in sera of patients suffering from colorectal cancer, especially in metastatic cases [115]. In two other reports, however, low levels of Gal-4 were associated with an advanced form of colorectal cancer [116,117], while stimulation of Gal-4 expression caused colorectal cancer cells to become sensitized to Camptothecin [117]. Conflicting results may be due to the presence of different Gal-4 isoforms that are not yet known. Evidently, further research is needed to clarify the function of Gal-4 and its role in cancer. Recently, there have been several studies which shed some light on the composition and function of Gal-4 [118,119,120]. Bum-Erdene et al. described, based on crystallography, the structure of the CRDs in relation with numerous ligands [119,120].

## 5. Galectin-7

Galectin-7 (Gal-7), was described for the first time in 1995 by Magnaldo et al. [121]. Initially considered as keratinocyte differentiation marker, Gal-7 is a prototype galectin capable of forming homodimers. Increased expression of Gal-7 was observed in numerous neoplasms, such as breast [122], thyroid [123] and throat [124] cancers, as well as in indolent lymphoproliferative diseases [125]. In more than one case, a correlation was identified between Gal-7 and the progression of neoplasms into more aggressive phenotypes [122,125]. Conversely, low expression of Gal-7 was observed in the case of gastric cancer [126], colon cancer [127], squamous cell carcinoma of the cervix [128] and urothelial bladder cancer [129]. Higher expression of Gal-7 in patients with squamous cell cervical cancer was associated with a better outcome after radiotherapy [130]. Labrie et al. observed increased Gal-7 expression when p53 was mutated [131]. Additionally, epithelial ovarian cancer cells secrete Gal-7, which through matrix metalloproteinase 9 (MMP-9) promotes invasiveness. In another study, mouse lymphoma cells transfected with an antisense Gal-7 plasmid showed reduced survival time [132]. We hypothesize that Gal-7 modulates *MMP-9* gene expression to some extent, since lymphoma cells transfected with Gal-7 antisense RNA, also showed a reduction in *MMP-9*. Based on crystallography of the Gal-7 molecule, a 2-*O*-galactoside benzyl phosphorane was synthesized. The new compound showed a 60-fold increased affinity for Gal-7 compared to galactoside [133]. Promising results were presented by Vladoiu et al., who used a selective inhibitor, hGal-7 to disrupt dimerization of Gal-7 and inhibit apoptosis of Jurkat T-cells [133]. This compound targets the dimer interface of Gal-7, but not at CRD. High concentrations of the drug were necessary to observe results and, thus, further studies are needed to improve on the molecule. A review article by Kaur and collaborators summarizes Gal-7 findings in cancer [134].

## 6. Galectin 8

The role of galectin 8 (Gal-8) in oncogenesis is not well understood. Gal-8 is a type of tandem-repeat galectin with two CRDs one at C- and another at N-terminal region joined by a polypeptide linker. The terminal domains are responsible for recognizing and binding ligands whereas the linking peptide regulates biological functions and it has a multimerization function [135]. Alternative splicing of the linker region results in the formation of a peptide of different length, which determines the formation of the various Gal-8 isoforms: Gal-8S (short liner region), Gal-8M (medium linker region) and Gal-8L (long linker region) [136,137]. These isoforms have different biological functions and can activate different signaling pathways, hence limiting the design of targeted therapies [138]. Gal-8 has been suggested to function in modulating angiogenesis [137]. Recent studies show that C-terminal CRD preferentially binds blood cell antigens A and B, as well as poly-LacNAc saccharides, while N-terminal CRDs have high affinity for sulfated and sialylated glycans [139,140]. In normal endothelial cells, Gal 8 binds CD166 [137] and CD44 [141]. Additionally, Gal-8 may be a useful marker of papillary thyroid cancer, where it is strongly expressed, unlike normal tissue where there is undetectable expression [142]. Loss of Gal-8 expression is associated with increased risk in urinary bladder cancer recurrence but not of tumor progression [143]. The expression of Gal-8 may be a potential predictor of early recurrence after nephrectomy in patients with localized pT1 clear cell renal cell carcinoma [144]. Additionally, Gal-8 is responsible for the progression of prostate cancer and initiation of metastatic phenotype [145]. Given that Gal-8 does is not expressed in healthy prostate tissue, it may be a potential therapeutic target in the future. Increased serum concentration of Gal-8 has been observed in breast cancer as well as colorectal cancer [146]. In breast cancer, Gal-8 expression was observed both intracellularly and extracellularly [147,148]. Satelli and colleagues hypothesized that intracellular Gal-8 undergoes post-translational processing representing the half-weight of the extracellular Gal-8 [148]. A recently published study presents the interactions between activated leukocyte cell adhesion molecule (ALCAM/CD166) and Gal-8, which may be important in the biology of breast cancer cells [149].

## 7. Galectin 9

Galectin 9 (Gal-9) was discovered and described for the first time in 1997 in patients suffering from Hodgkin’s lymphoma (HL) [150]. Gal-9 is a type of tandem-repeat galectin with 2 CRDs, a 148-amino acid-long N-terminus and a 149-amino acid-long C-terminus. Between domains there is a connecting sequence, whose length determines the three isoforms of Gal-9: short Gal-9S, medium Gal-9M and long Gal-9L. The three isoforms exhibit varying degrees of chemotactic effects on eosinophils [151]. Gal-9 is present in both intracellular and extracellular compartments [11] and several ligands of Gal-9 have been described. Extracellular Gal-9 can bind to TIM-3, CD44 and Glut-2, while intracellular Gal-9 binds transcription factor NF-IL6 [152,153,154,155]. Gal-9 has been described to play an important role in numerous biological processes such as adhesion [156], aggregation of cancer cells [157], apoptosis [158,159], immunomodulation [160,161] and chemotaxis [162]. In most cases, Gal-9 expression in healthy tissues is higher than in neoplastic cells as observed in breast [157], liver [162], lung [163], prostate [164], kidney cancers [163] and melanoma [156]. Increased Gal-9 expression reflects progression and aggressiveness of the neoplasm. In a few cases, high Gal-9 expression was described in Hodgkin’s lymphoma [150], colorectal [163], oral [165] and pancreatic cancer [166]. Differences in Gal-9 expression are caused by differential mRNA splicing and generation of different isoforms.

Gal-9 is a good prognostic factor in patients who suffer from renal cell carcinoma (RCC) [167]. High Gal-9 expression is associated with decreased overall survival (OS) time and decreased recurrence free survival (RFS). Patients with high Gal-9 expression showed more advanced progression of the disease with larger tumor size and necrosis [168]. Interestingly, Gal-9 proved ineffective in the stratification of patients with advanced disease (TNM III/IV, Fuhrman 3/4). Moreover, patients suffering from metastatic RCC, who had responded to IL-2 and IFN-γ therapy, showed high Gal-9 expression [168]. Unlike other galectins, Gal-9 predominantly functions as tumor suppressor. Studies performed in hepatocellular carcinoma (HCC) cell models indicated that silencing of Gal-9, by small interfering RNAs (siRNA), resulted in increased proliferation and migration [162]. Additionally, patients with positive Gal-9 expression had longer survival times than those with negative lesions [135].

A study by Wiersma and collaborators, showed that a recombinant form of Gal-9 was shown to promote cell death in colorectal carcinoma cells (CRCC) with KRAS mutations. CRCCs are commonly resistant to chemotherapy and immunotherapy when EGFR inhibitors are used. Following administration, recombinant soluble Gal-9 (rLGALS9) rapidly entered cells by endocytosis and accumulated in lysosomes. Internalization of rLGALS9 resulted in autophagosome–lysosome fusion failure, lysosome swelling, accumulation of autophagosomes and ultimately cell death [169]. Inhibition of the autophagosome-lysosome fusion was described earlier following the application of the lysosomal inhibitor, chloroquine [170]. Conversely, rLGALS9 therapy on CRC cells with the BRAF mutation caused no effects [169].

In another study, chronic myeloid leukemia (CML) cell lines resistant to tyrosine kinase inhibitors (TKIs) were treated with modified human Gal-9 (hGal-9). Resistance of cells to treatment was overcome and a synergistic activity of hGal-9 with TKIs was noted. The process of apoptosis occurred through the activation of activating transcription factor-3–Noxa proapoptotoic pathway (ATF3–Noxa) and was independent of p53 expression. In this study, hGal-9 was also reported to activate caspase-4 and caspase-8 through a TKI-independent pathway [171]. Interestingly, the Tim-3/Gal-9 signaling pathway is described as a one of the immune checkpoints responsible for T-cell exhaustion [172]. Inhibition of this signaling pathway may be an important therapeutic option in cancer patients. However, we now know that Gal-9 also has other membrane receptors on the surface of T cells [173] and that Tim-3 receptor has non-Gal-9 ligands [174]. A recent excellent review by Riayo Yang and Mien-Chie Hung summarizes the current research on the role of Tim-3 and Gal-9 in antitumor immunity [172].

## 8. Other Galectins

There are 16 types of galectins, however, the role of other Galectins has not been fully understood nor documented. In a recent study by Peng and collaborators, IHC staining showed increased levels of Gal-10 protein in all stages of colorectal carcinoma progression [175]. In another study, Gal-12 protein levels were found to be increased in a public data set of 526 acute myeloid leukemia (AML) samples of various FAB subtypes especially the M3 subtype [176]. Current research and publications knowledge is expected to be broadened soon.

## 9. Conclusions

In this review, the scientific evidence obtained through extensive study of galectins suggests that inhibition of galectin activity may contribute to better antineoplastic drugs. Preclinical and clinical studies indicate that inhibiting galectin action results in tumor growth arrest, inhibition of angiogenesis and occurrence of metastases. Additionally, administration of galectin inhibitors in combination with chemotherapy and radiotherapy improves efficacy of treatment of various neoplasms. Furthermore, inhibition of galectins has been shown to interfere with multidrug resistance mechanisms (MDR) enhancing sensitivity of chemotherapeutics. An important aspect to take into account is that tumor cells can express more than one galectin, and therefore, tumor-specific therapy is crucial for therapeutic benefit. Unfortunately, most research is restricted to cell lines and animal models and results from early phase human trials have been inconclusive. In colon cancer, tumor cells have increased expression of Gal-1, -3, -7, and -10, which correlated with increased blood levels of Gal-1, -2, -3, -4, -8, and -9 [1]. Another level of complexity in the design of novel therapies relies on the fact that galectins can present in various isoforms, of which only some affect the function of cancer cells. Additionally, tumor-clonal expansion may lead to the production of a tumor which may not express the expected galectin, hindering the discovery of targeted therapy. Another aspect to take into account in the design of therapeutic inhibitors is the balance between intracellular and extracellular localization of galectins. Current inhibitors only block extracellular functions of a given galectin and neglect intracellular functions as seen in Gal-1 and Gal-3. Consequently, further research is warranted to assess the role of galectins in cancer therapy. Nowadays, several clinical trials are being conducted focused on the use of galectins in the treatment of neoplasms (Table 4). The use of an inhibitor for a single galectin could be effective, provided that selectivity is adequate and that the above-mentioned problems are taken into account before clinical trials commence. However, as research progresses, galectin targeting therapy may increase the efficacy of cancer patient treatment.

## Figures and Tables

**Table 1:** Galectin characteristics according to molecular structure.

Subtype	Galectins	Model	Structure
Prototype	1, 2, 5, 7, 10, 11, 13, 14, 15, 16	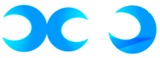	Each monomer of homodimer contains CRD
Tandem Repeats	4, 6, 8, 9, 12	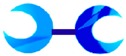	Two CRD domains connected with linker
Chimeric	3	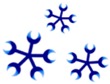	Multimeric structures with one CRD in C terminus and N-terminus.

**Table 2:** Galectin-1 inhibitors.

Inhibitor	Target	Effect	Refs.
Thiodigalactoside (TDG)	Melanoma and breast cancer xenografts; Colon and breast cancer xenografts	Induction of apoptosis; Inhibition of angiogenesis, proliferation and tumor growth; Reduction of lung metastases; Restore T cells surveillance	[30,31]
Anginex (β pep-25)	Ovarian, melanoma and breast cancer xenografts	Inhibition of tumor growth, angiogenesis and migration; Increased sensitivity to radiotherapy and chemotherapy; Synergistic effects with bevacizumab	[33,34,35,36]
6DBF7; DB16; DB21	Lung and ovarian cancer and melanoma xenografts	Inhibition of tumor growth; Inhibition of angiogenesis	[37,38]
OTX008 (0018)	Ovarian cancer xenografts; Head and neck and ovarian cancer cell lines; Clinical trial in patients with advanced solid tumors	Downregulation of cancer cell proliferation; Inhibition of tumor growth, angiogenesis and migration; Synergic effects with chemo- and immunotherapy	[24,39,40,41,42]
F8.G7	Endothelial cells; Kaposi’s sarcoma xenografts	Inhibition of tumor growth, angiogenesis, migration	[43,44]
GM-CT-01 (DAVANAT^®^) oraz GR-MD-02	Colon cancer xenografts; Clinical trials in patients with colon cancer and melanoma	Inhibition of tumor growth; Restore the T cells surveillance	[45,46]

**Table 3:** Galectin-3 inhibitors.

Inhibitor	Target	Effect	Ref.
G3–C12	Breast, colon and prostate cancer xenografts	Reduction of lung metastasis; Induction of apoptosis; Inhibition of tumor growth; Synergic effect with chemotherapy	[93,94,95,96]
Modified citrus pectin (MCP)	Breast and colon cancer xenografts; Prostate cancer cell lines; Patients with advance solid tumors	Inhibition of tumor growth, angiogenesis and metastasis; Induction of apoptosis; Cell cycle arrest; Increase sensitivity to chemotherapy; Rebalance the T cells surveillance	[98,99,100,101,102]
PectaSol-C Modified citrus pectin	Prostate and ovarian cancer cell lines	Induction of apoptosis; Inhibition of proliferation; Synergic effect with chemotherapy	[98,103,104]
GCS-100	Multiple myeloma, DLBCL cell lines; Prostate cancer cell lines; Patients with Chronic lymphocytic leukemia (CLL)	Inhibition of cell growth; Induction of apoptosis; Synergic effect with chemotherapy; Increased sesnsitivity to immunochemotherapy	[92,105,106,107,108,109]

**Table 4:** Ongoing clinical trials with galectin inhibitors in oncology.

NCT Number	Inhibitor	Target	Phase	Title of the Study
NCT01723813	GM-CT-01	Gal-3	I/II	Peptide Vaccinations Plus GM-CT-01 in Melanoma
NCT01724320	OTX008	Gal-1	I	A Phase I, First-in-man Study of OTX008 Given Subcutaneously as a Single Agent to Patients with Advanced Solid Tumors
NCT02117362	GR-MD-02	Gal-3	I	Galectin Inhibitor (GR-MD-02) and Ipilimumab in Patients with Metastatic Melanoma
NCT02575404	GR-MD-02	Gal-3	I	GR-MD-02 Plus Pembrolizumab in Melanoma Patients
NCT01681823	PectaSol-C	Gal-3	III	Effect of Modified Citrus Pectin on PSA Kinetics in Biochemical Relapsed PC with Serial Increases in PSA

Data were collected from: https://clinicaltrials.gov.
